# Global protein sustainability and the United Nations, through to the 2030 agenda

**DOI:** 10.3389/fnut.2024.1383898

**Published:** 2024-10-15

**Authors:** Barbara Burlingame, Ana Moltedo, Carlo Cafiero

**Affiliations:** ^1^Riddet Institute, Massey University, Palmerston North, New Zealand; ^2^Food and Agriculture Organization of the United Nations, Rome, Italy

**Keywords:** protein, United Nations, policy, nutrition, FAOSTAT, Food and Agriculture Organization of the United Nations

## Abstract

Organizations and initiatives concerned with food security and nutrition have long positioned protein, together with dietary energy, as the keystone for life itself. Indeed, the word protein, derived from the Greek *proteios*, means ‘of primary importance’. There is a long history of attention to, and controversies over, proteins in UN processes, beginning in the 1930s and continuing to this day. The importance of protein for agriculture, health, food security and nutrition is reflected in the data collected and presented in the statistical databases of the Food and Agriculture Organization (FAOSTAT), available per commodity, per country and over an extensive time series. Protein features directly and indirectly in all 17 Sustainable Development Goals (SDG), which constitute the United Nations 2030 Agenda. Most directly involved is SDG 2. The short title for SDG 2 is ‘zero hunger’. The long title offers more detail: end hunger, achieve food security and improved nutrition and promote sustainable agriculture.

## Introduction

### Historical overview of protein and the UN

International cooperation and collaboration in nutrition began in earnest in 1936 when the League of Nations set up a technical committee to establish recommended levels of protein intake (1). In 1945, with the creation of the United Nations (UN) and soon thereafter its specialized technical agencies, attention to protein continued. Several of the UN’s specialized agencies concerned themselves with dietary protein, but the two with the longest history of dealing specifically with proteins are the Food and Agriculture Organization of the United Nations (FAO) (2), and the World Health Organization (WHO) (3). Of major concern was the ‘protein gap’ related to both production and consumption (4). In one way or another, the theoretical protein gap and its remedies feature directly and peripherally in goals, targets, policies, research, interventions, and more, to this very day.

In 1948–1950, FAO established and convened meetings of the Standing Advisory Committee (5) to address the most pressing nutrition problems, with protein and dietary energy at the top of the list. From the late 1940s, there was a series of meetings and several technical reports on protein (2, 3), as it was commonly agreed that a major nutrition problem was a lack of sufficient protein in the diets of young children, known as kwashiorkor from the Ga language of Ghana (6). The First Joint FAO/WHO Expert Committee on Nutrition noted that “one of the most widespread nutritional disorders in tropical and sub-tropical areas is the syndrome at present ill-defined and known by various names such as kwashiorkor” (7).

In 1971, the UN itself in the body of the General Assembly (UNGA) devoted a full segment of its meeting to protein resources (8, 9). The membership put forward a set of 16 resolutions as “Essential elements of the Strategy Statement on Action to Avert the Protein Crisis in the Developing Countries.”

Viewed from a 21st century vantage point, some of these protein-related resolutions succeeded, while others remain intransigent 50+ years later and feature in the goals and targets of the 2030 Agenda. Table 1 shows a subset of the resolutions mapped to comparable SDGs, along with comments on the success, failures, and consequences over the timeframe. Among the resolutions identified as successful are some that ironically also contribute to our sustainability crises, with specific examples. On several occasions since then, the UNGA has returned its focus to protein, mainly in the context of livestock, climate change and consumption of animal source proteins (10).

**Table 1:** Comparing the 1971 United Nations General Assembly (UNGA) 26th session recommendations on protein resources (1) with relevant/comparable Sustainable Development Goal (SDG) targets (2).

UNGA 26th Protein Session, 1971	SDG targets	Notes/comments
Make every effort to increase the production of food crops, particularly through the exploitation of new high-yield varieties, bearing in mind the special need for an expanded production of protein-rich pulses and oilseeds;	2.3 “Double the agricultural productivity and incomes of small-scale food producers, in particular women, indigenous peoples, family farmers, pastoralists and fishers….”	New varieties and high yield are the UNGA focus, as the Green Revolution is seen as a great agriculture success story. The specific focus on pulses and oilseeds is particularly noteworthy. Implicit is encouragement for the movement away from too-heavy reliance on animal source foods, which is a hallmark of many sustainability recommendations (3). Unfortunately, the SDGs make no mention of pulses or other high protein plant source foods.
Encourage accelerated and expanded research designed to improve the nutritive value of cereal proteins through genetic engineering;	2.5 “Maintain the genetic diversity of seeds, cultivated plants and…their related wild species ….”	UNGA, at the time exposed to primarily only to the benefits of the Green Revolution, does not consider biodiversity (genetic diversity) in its recommendations. The SDGs, 40 years later and therefore mindful of the negative consequences, focus instead on conserving biodiversity.
Encourage accelerated and expanded research designed to develop high-yielding pulses, legumes and oilseed crops;	2.4 “Ensure sustainable food production systems and implement resilient agricultural practices that increase productivity and production, that help maintain ecosystems, ….”	As above, UNGA valued food crops engineered for high yield to the exclusion of many other considerations. Explicit in the complete text for SDG 2.4 is “the need to improve ecosystems, that strengthen capacity for adaptation to climate change, extreme weather, drought, flooding and other disasters and that progressively improve land and soil quality.”
Encourage the increased production of animal proteins, particularly through research on increasing forage yields and production;	2.5 “Maintain the genetic diversity of… farmed and domesticated animals and their related wild species…and promote access to and fair and equitable sharing of benefits arising from the utilization of genetic resources and associated traditional knowledge ….”	UNGA references animal production for human nutrition, whereas the SDGs avoid livestock-related targets for both production and consumption. Nevertheless, animals are included in the ‘no hunger’ goal, but seemingly for their function as ecosystems services.
Make every effort to prevent an unnecessary loss of protein-containing foods in field, storage, transport and home	12.3 “Halve *per capita* global food waste at the retail and consumer levels and reduce food losses along production and supply chains, including post-harvest losses…”	Unacceptably high levels of food losses and waste remain an enduring problem and appear with equal consideration in both the SDGs and UNGA recommendations.
Encourage increased production from marine and freshwater fishery resources	14.4 “Effectively regulate harvesting and end overfishing, illegal, unreported and unregulated fishing and destructive fishing practices and implement science-based management plans, in order to restore fish stocks in the shortest time feasible, at least to levels that can produce maximum sustainable yield as determined by their biological characteristics…”	It was obviously not foreseen in the UNGA recommendation that production from fishery resources would quadruple over the next 50 years to become unsustainable.
Conduct informational and educational campaigns related to protein production and consumption	12.8 “Ensure that people everywhere have the relevant information and awareness for sustainable development and lifestyles in harmony with nature…”	
Improve protein utilization through the control and prevention of infectious diseases	2.2 “End all forms of malnutrition, including achieving, by 2025, the internationally agreed targets on stunting and wasting in children under 5 years of age ….”	Given that stunting in particular is often a manifestation of insufficient quantity or poor quality protein, SDG 2.2 is well aligned to the UNGA recommendation.
Review and improve policies, legislation and regulations regarding all aspects of food and protein production, processing and marketing so as to remove unnecessary obstacles and encourage appropriate activities	2.b “Correct and prevent trade restrictions and distortions in world agricultural markets, ….” 2.c “Adopt measures to ensure the proper functioning of food commodity markets and their derivatives ….”	The UNGA recommendation and SDG 2b, 2c are well aligned, acknowledging the obvious technological and regulatory developments over time.
Give special attention to the protein needs of vulnerable groups; and Initiate intervention programmes aimed at ensuring that vulnerable groups will receive the most appropriate type and a sufficient quantity of food by the most effective means	2.1 “End hunger and ensure access by all people, in particular the poor and people in vulnerable situations, including infants, to safe, nutritious and sufficient food all year round…”	Considering that 40+ years after UNGA 1971, and equally 40 years before, SDG 2.1 has been the most basic enduring problem for humankind, with hunger representing the poignant denial of the most basic of human rights.
Recognize the role of economic development and social modernization in solving the protein problem.	12.8 “Ensure that people everywhere have the relevant information and awareness for sustainable development and lifestyles in harmony with nature…”	One of the greatest shifts in thinking from the time of the UNGA recommendations to the time of the SDGs is the recognition that ‘economic development and social modernization’ is often in conflict with ‘harmony with nature’. Throughout the SDG, harmony with nature takes priority.

1. UN General Assembly. Protein Resources. New York: UN General Assembly 26th Session; December 20, 1971 p. 68–70.

2. United Nations. The UN Sustainable Development Goals. New York; 2015.

3. Jones R, Vogliano C, Burlingame B. Sustainable diets and food-based dietary guidelines. In: Burlingame B, Dernini S, editors. Sustainable diets: linking nutrition and food systems. CABI; 2018. p. 158–71.

In the 1972 meeting of the UN Protein Advisory Group (9), Hugues Gounelle de Pontanel states the conclusion that, “*Every doctor, nutritionist or political leader concerned with the problem of world hunger has now concluded that the major problem is one of protein malnutrition.”*

But not everyone agreed, and one of the most spectacular controversies in nutrition – the Great Protein Fiasco—became public soon thereafter (11) (see below). Nevertheless, from the 1970s to the present, FAO and WHO, and occasionally with the International Atomic Energy Agency and United Nations University, conducted many more meetings and expert consultations leading to protein-related reports, policies and recommendations, and necessary reviews and revisions as nutrition science and data availability increased and improved over time. Topics included protein requirements, production issues, measurement/methods, composition/quality, and consumption. Table 2 provides a list.

**Table 2:** Examples of protein reports from United Nations (UN) agencies, 1936 to the present.

1936: Report on the Physiological Bases of Nutrition. The Health Committee of the League of Nations, Geneva.
1949. Report of the First Joint FAO/WHO Expert Committee on Nutrition. Geneva, 24–28 October. Geneva.
1957: Protein requirements: report of the FAO Committee. FAO Nutritional Series No. 16, 1957
1963: Protein requirements - Report of a Joint FAO/WHO Expert Group. FAO Nutrition Meeting Report Series No. 37, 1964 and WHO Technical Report Series No. 301, 1964.
1970: Amino-Acid content of foods and biological data on proteins. FAO food and nutrition series. Rome, Italy: FAO.
1973: Energy and protein requirements: Report of a joint FAO/WHO *ad hoc* expert committee. Rome: FAO Nutrition Meetings Report Series No. 52. Geneva: WHO Technical Report Series No. 522.
1974: ‘The Great Protein Fiasco’; McLaren calls the protein gap theory “one of the greatest errors committed in the name of nutrition science (11).”
1981: Joint FAO/WHO/UNU Expert Consultation on Energy and Protein Requirements
1985: WHO/World Health Organization/Food and Agriculture Organization/United Nations University (1985) Energy and protein requirements Report of a Joint FAO/WHO/UNU Expert Consultation. WHO Technical Report Series, No. 724. Geneva: WHO.
1991: Protein quality evaluation. Report of Joint FAO/WHO Expert Consultation, FAO Food and Nutrition Paper 51.
2007: Protein and Amino Acid Requirements in Human Nutrition Report of a Joint WHO/FAO/UNU Expert Consultation. WHO Technical Report Series No. 935
2013: Dietary protein quality evaluation in human nutrition: Report of an FAO Expert Consultation, FAO Food and Nutrition Paper 92.
2019: Nitrogen and protein content measurement and nitrogen to protein conversion factors for dairy and soy protein based foods: a systematic review and modeling analysis. WHO and FAO.

## Assessment and implications

### The great protein fiasco

The UN reports and their recommendations were not without controversy (11). World protein supply has long been estimated using production data from FAO and national statistical agencies, consumption data made mainly with proxy measures from FAO food balances (i.e., protein available for human consumption) and disappearance data, which is then analyzed against requirements (without considering inequalities in accessing protein in the population). As such, there seemed to be a shortfall which was called the “protein gap.” Consideration was also given to protein quality measurements and calculations, further defining the gap, as vegetable and other non-animal-source proteins were of poorer quality than animal source proteins. It was concluded by scientists and policy-makers alike that the protein gap would only widen unless alternative or unconventional sources of high-quality protein could be found. Leaf protein concentrate, insects and single cell organisms (12) were then included in nutrition research and development programmes around the world. The Protein Advisory Group, a UN agency, had been established in 1955 to advise on the “safety and suitability” of these new protein-rich foods.

However, in 1974, Donald McLaren, professor at the American University in Beirut, published a paper in The Lancet titled: “The great protein fiasco” (11), proposing that dietary energy should be the focus of attention, and that would bring about adequacy across the nutrient spectrum, protein included. A year after McLaren’s paper appeared in The Lancet, John Waterlow and Philip Payne from the London School of Hygiene and Tropical Medicine published an analysis of diets of children in developing countries (13). Their analysis revealed that protein deficiency was rare, and when it occurred it was caused by a simple lack of food, rather than the low-protein content of food. In a 2011 interview, reflecting on his life and career, McLaren described the belief in the protein gap as “one of the greatest errors committed in the name of nutrition science in the past half-century” (14). Was it, though? Debates continue, with the overriding view that there is a nutrition crisis in the world, with protein as a feature, and it is related to both production and consumption.

### UN’s 2030 agenda

SDG 2, the hunger goal, has five targets, 2.1–2.5, with an additional three added (2.A, 2.B, 2.C); each target has one or more indicator(s). The following section presents data from the FAO Statistical Databases (FAOSTAT), some of which correspond to SDG indicators, showing trend analyses and projections into the future. The data on protein available for human consumption used in this study are derived from the latest series of Food Balance Sheets (FBS) based on a new methodology (15) and from the new dataset of nutrient conversion factors (16), both developed by FAO. Data expressed in units ‘*per capita* per day’ reflect availability within a country/region or special group and are used as a convenient but crude proxy for consumption.

**Figure 1:**
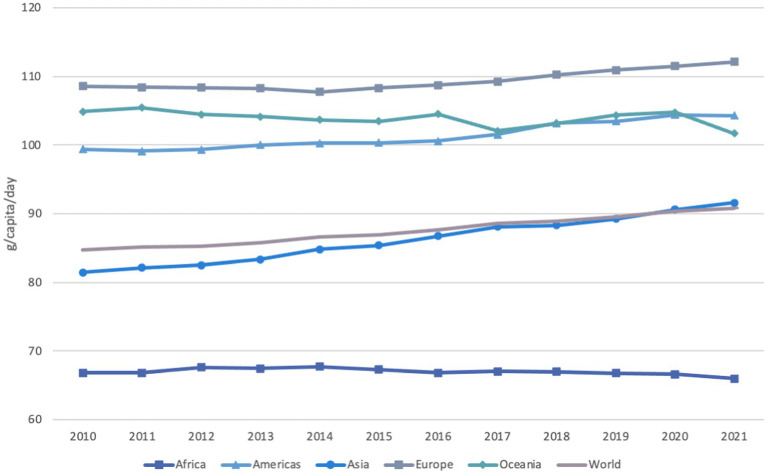
Total dietary protein supply by region and in the world between 2010 and 2021, in g/capita/day. Reproduced from FAO. 2023. FAOSTAT. Food Balances. Food Balances (2010-), licensed under CC BY 4.0.

Figure 1 shows that dietary protein available for human consumption at the global level has increased by 7 % since 2010, from around 85 to more than 90 g/capita/day, despite slight decreases in Africa and Oceania. Europe has the highest dietary protein supply (112 g/capita/day) in 2021, followed by the Americas (104 g/capita/day), Oceania (102 g/capita/day), Asia (92 g/capita/day) and Africa (66 g/capita/day). Protein quality is not considered in this metric.

**Figure 2:**
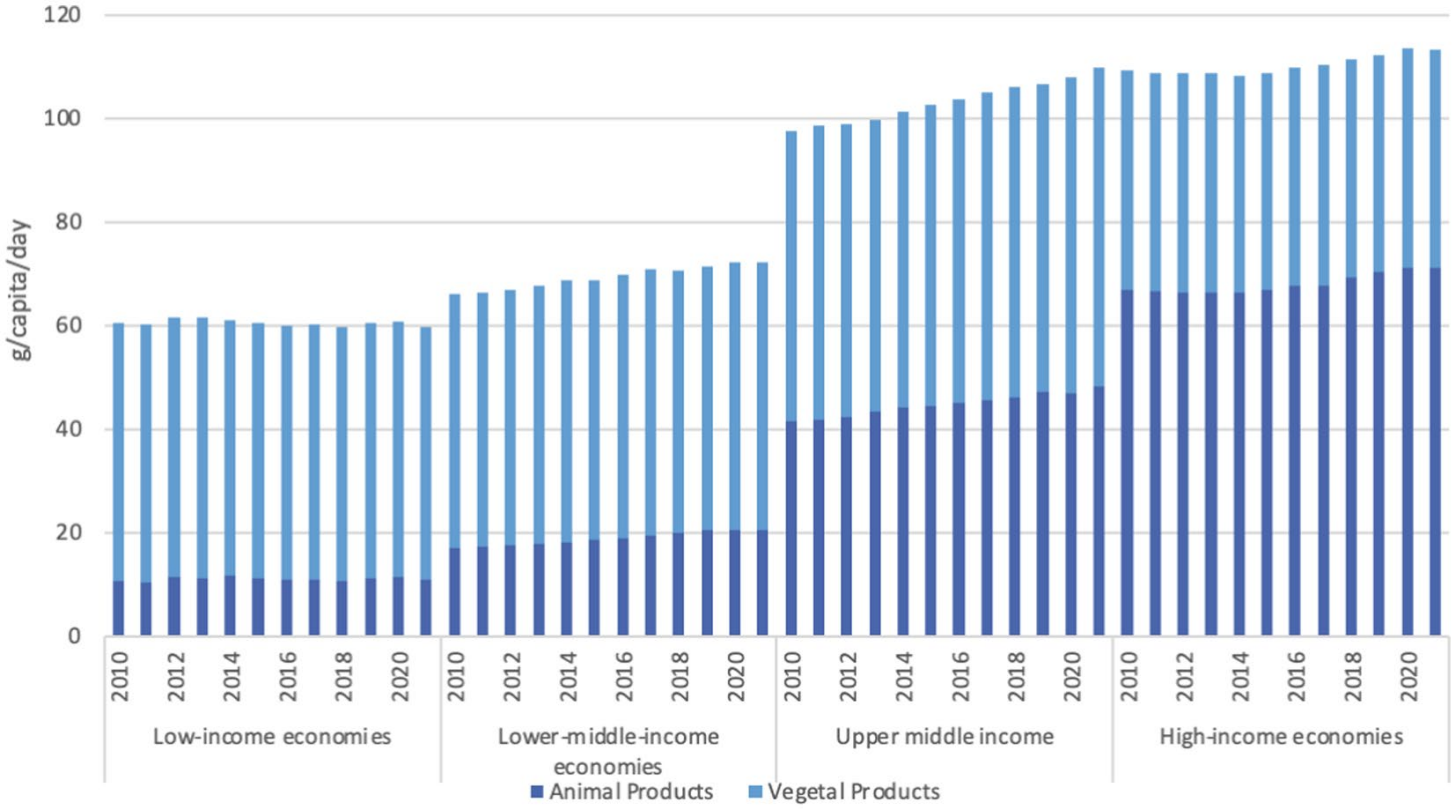
Dietary protein supply from animal and vegetal sources by income economy, in g/capita/day. Reproduced from FAO. 2023. FAOSTAT. Food Balances. Food Balances (2010-), licensed under CC BY 4.0.

Both animal and vegetal foods supply dietary protein, with animal source foods providing higher quality protein than vegetal foods (based on quantity and balance in the amino acid composition). As shown in Figure 2, the proportion supplied by each source of protein varies with the income level of the country (17). In 2021, in high-income countries, 63 percent of the protein (amounting at 71.2 g/capita/day) is supplied from animal sources. In low-income countries it was only 18 percent (amounting at 10.9 g/capita/day). Controversies abound regarding the conflicting issues surrounding livestock production and consumption – nutritional equity, or lack thereof which is illustrated with these data, plus human health and environmental sustainability risks and benefits, to name but a few.

**Figure 3:**
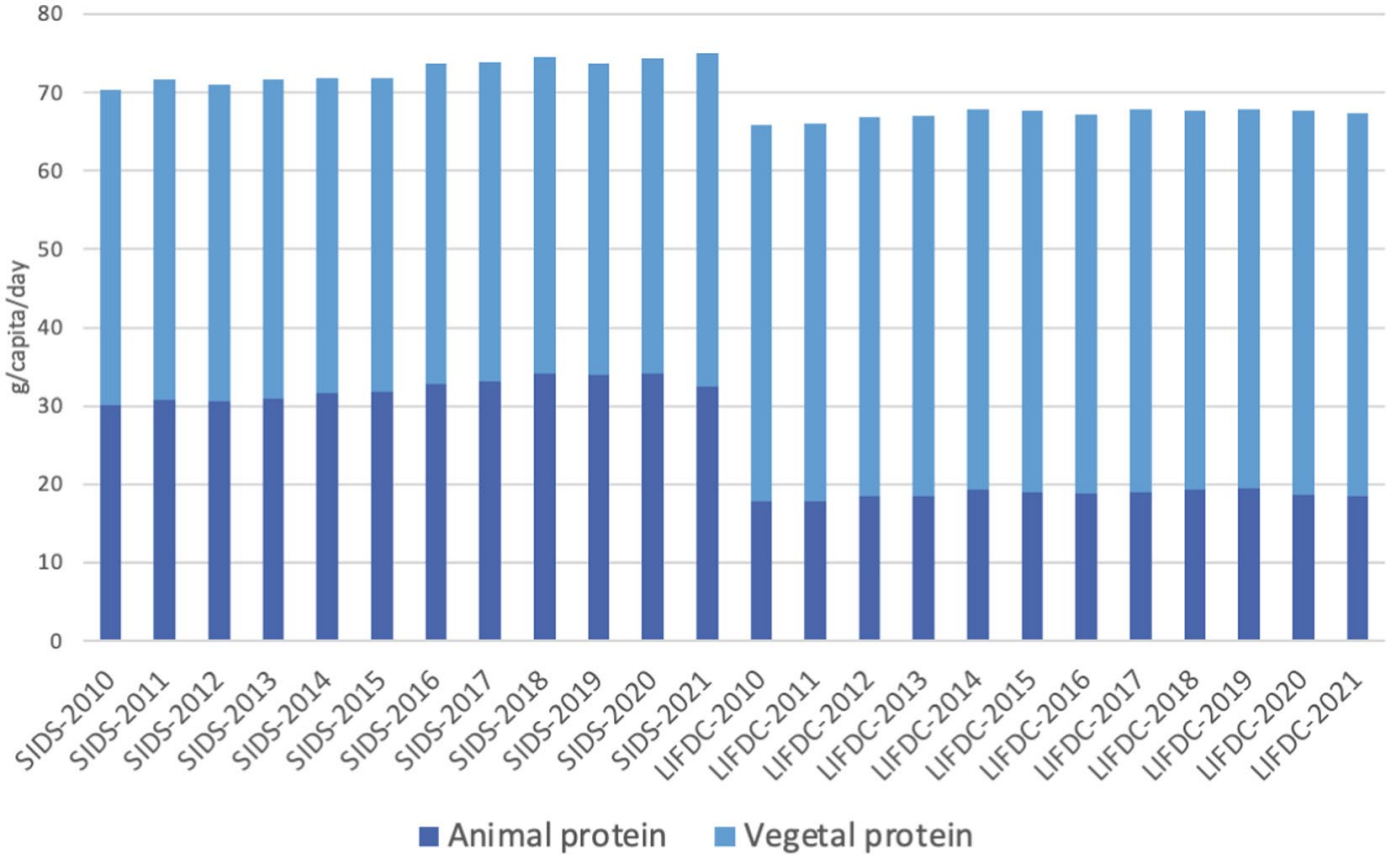
Dietary protein supply from animal and vegetal sources for special groups, in g/capita/day. Notes: SIDS, Small Island Developing States; NFIDC, Net Food Importing Developing Countries. Reproduced from FAO. 2023b. FAOSTAT. Food Balances. Food Balances (2010-). November 2023, licensed under CC BY 4.0.

Focusing on special groups, in 2021 the percentage of protein from animal sources in small islands developing states was 43 percent, equivalent to 32.4 g/capita/day. On the contrary, that year, the net food importing developing countries obtained 27 percent of protein from animal sources equivalent to 18.4 g/capita/day (Figure 3).

**Figure 4:**
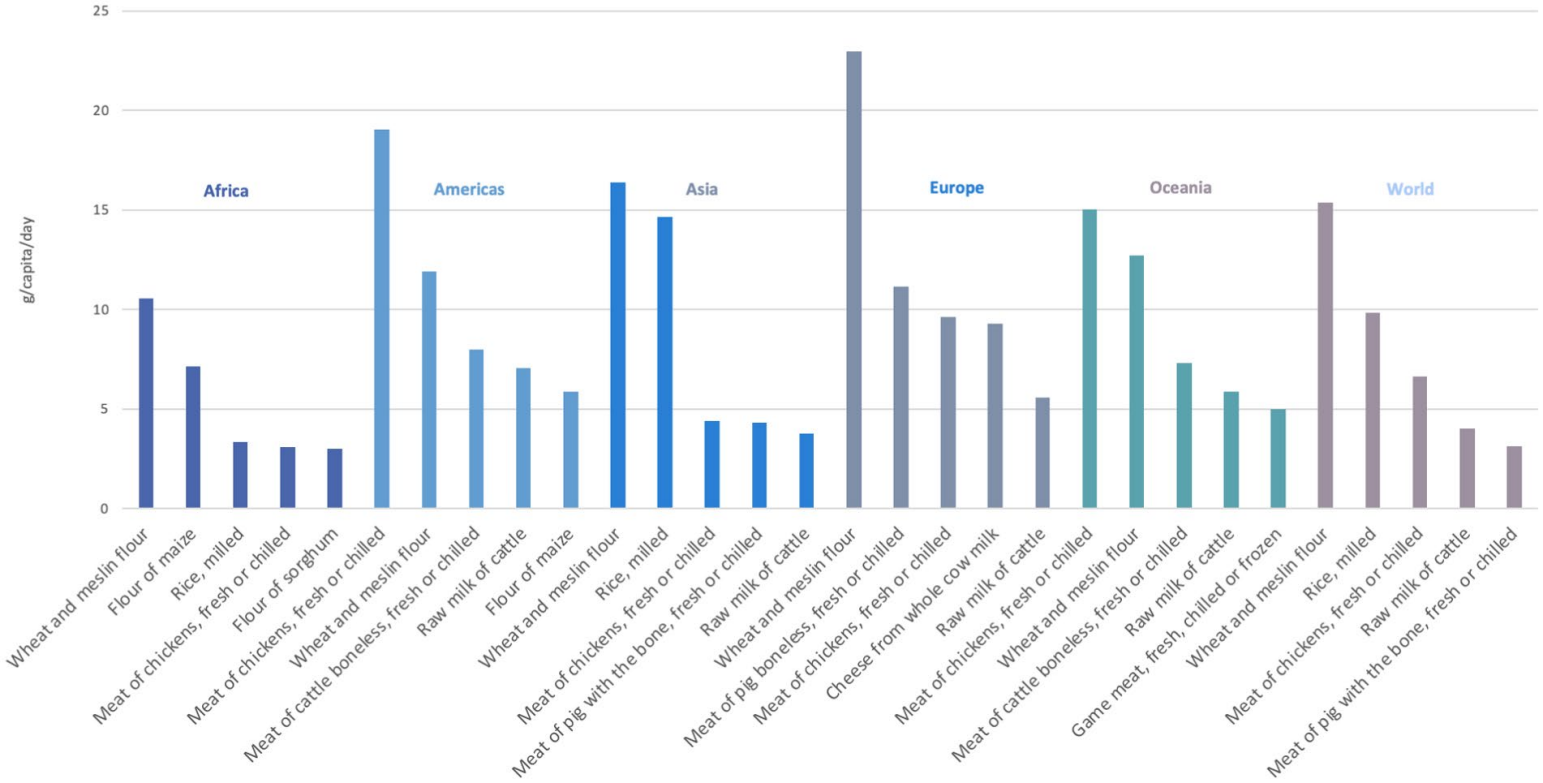
Top five main food sources of dietary protein by region and in the world, in 2021, in g/capita/day. Reproduced from FAO. 2023. FAOSTAT. Food Balances. Supply Utilization Accounts (2010-) [on the internet], licensed under CC BY 4.0. Population and Employment. Annual Population [on the internet], licensed under CC BY 4.0.

Figure 4 shows the top five providers of protein from crops and livestock, in 2021. Wheat flour was the main source of protein in the world, and in Africa, Asia and Europe, while it was chicken meat in the Americas and in Oceania. Among animal food, chicken meat is the main source of protein everywhere except Europe and Asia, where pig meat is the main source. Other relevant sources are cattle meat in the Americas, chicken meat in Asia and pig meat in Oceania. Raw milk of cattle was within the top five main sources in all regions except Africa, where four out of the five main sources were cereals. Raw milk of cattle + meat was within the top five providers of protein in all the regions.

**Figure 5:**
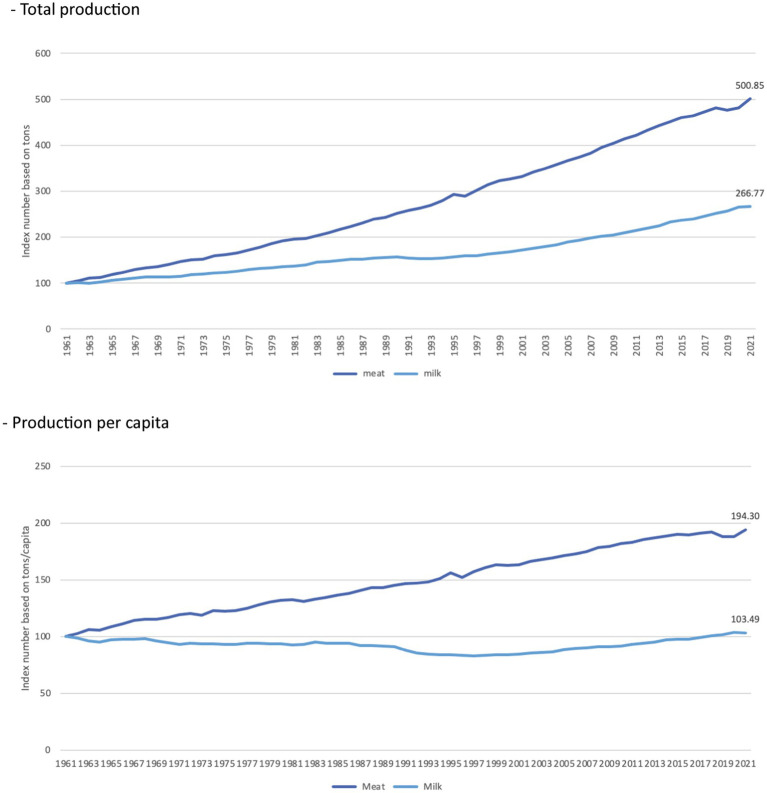
Index numbers of the production of meat and milk in the world between 1961 and 2021 (1961 = 100). Note: Production of raw milk (from all livestock including the amount used to feed them); Production of meat (red and white meat from commercial and farm slaughter) is given in terms of dressed carcass weight, excluding offal and slaughter fats. Reproduced from FAO. 2023. FAOSTAT. Production. Crops and livestock products [on the internet], licensed under CC BY 4.0.

As presented in Figure 5, since 1961, in the world, the production of milk (from all livestock including the amount used to feed them) and meat (in terms of dressed carcass weight, excluding offal and slaughter fats) have increased (16); however, at different paces. While the production of meat has increased five-fold since 1961, that of milk increased only by a factor of 2.7. In *per capita* terms (18), the global production of meat has almost doubled since 1961, while that of milk remained fairly constant.

## Discussion and recommendations

Presented here is a brief review of UN-led evidence-based initiatives on protein, along production and consumption data from FAOSTAT, the combination of which forms the foundation of policies and programmes, and indeed, the part of the SDG monitoring. But the current activities and data are only small pieces for a bigger puzzle requiring integration of many sectors and disciplines. Looking back at some of the recommendations from the 1971 UNGA meeting (8), it should have been predictable that ‘increased production of animal proteins, particularly through research on increasing forage yields and production’ could lead to environmental degradation and biodiversity loss; or that ‘increased production from marine and freshwater fishery resources’ could lead to crises in the capture fisheries sector with over-fishing, and extreme pollution from the farmed fish sector. Similarly, what are the consequences of our current trajectory for protein production, consumption, and research?

FAOSTAT provides useful data on consumption and production for national and global assessments, and monitoring trends over time, but more granularity and greater disaggregation would improve the value of these data sets for achieving goals and targets related to human nutrition generally, and protein specifically. It is clear, despite all the efforts and initiatives focussing on human nutrition, that the world is not on track for meeting the 2030 Agenda. In the time remaining, different forms of knowledge, including traditional knowledge from the millennia of lived science of indigenous peoples, need to be given greater attention, and more transdisciplinary and multisectoral collaborations need to be marshaled for sustainable development to be a reality. This includes the realm of protein, resolving and/or avoiding another protein fiasco.
